# Supplementary material for: Large non-thermal contribution to picosecond strain pulse generation using the photo-induced phase transition in VO2
Source: Nat Commun. 2020 Apr 3;11:1690. doi: 10.1038/s41467-020-15372-z (PMC7125085; doi:10.1038/s41467-020-15372-z)
Supplement: Supplementary file 1 — Supplementary Information [file 41467_2020_15372_MOESM1_ESM.pdf]

Supplementary Information

Large non-thermal contribution to picosecond strain pulse generation using the photo-induced phase transition in VO_2

Mogunov et al.

Supplementary Note 1: Samples and crystallographic data

Two epitaxial VO_2 films were grown by pulsed laser deposition (PLD) technique on single-crystal 290 μm-thick r-cut (012) Al_2O_3 substrates. PLD growth was performed in an oxygen and argon atmosphere with total chamber pressure of 30 mTorr, at 550 °C substrate temperature. A metallic vanadium target was ablated by excimer KrF laser pulses (20 ns duration, 248 nm wavelength with 4 J cm^{-2} fluence). The details of the growth procedure can be found in [1].

The sample structure and morphology was verified by X-ray diffraction (XRD) using a Bruker D8 Discover diffractometer, performing θ-2θ scans and azimuthal measurements. For 100 nm and 35 nm VO_2 films [Supplementary Figure 1], the XRD data show a single monoclinic M1 phase with (100) planes parallel to the substrate surface, as is indicated by the (200) VO_2 diffraction peak at 2θ=37.1° for both films. No other vanadium oxide phases were detected. Other observed peaks, (012)Al_2O_3, (024)Al_2O_3, and CuK_β radiation repeats of the same peaks correspond to XRD from the Al_2O_3 (r-cut) crystal substrate.

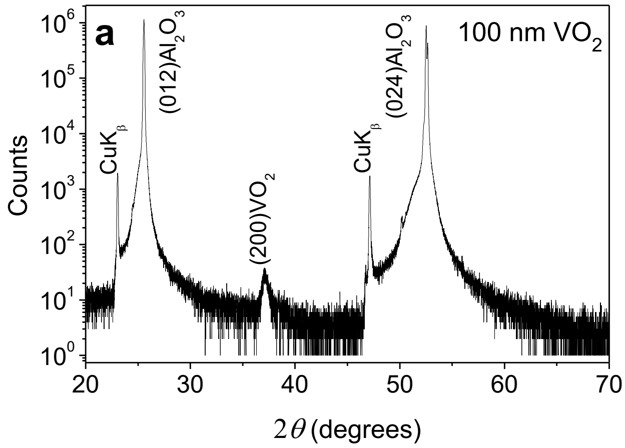

Supplementary Figure 1. Room-temperature X-ray diffraction for (**a**) 100 nm VO_2 film and (**b**) 35 nm VO_2 film grown on the r-cut Al_2O_3 substrate.

To identify the in-plane orientation of VO_2 lattice on Al_2O_3 (r-cut) substrate, a set of azimuthal XRD measurements were performed. Thus, we found (002)VO_2, (011)VO_2 and (006)Al_2O_3 Bragg reflections at expected inclination angles of the goniometer χ=57.40°, χ=67.63° and χ=57.61°, respectively [Supplementary Figure 2]. For all the studied VO_2/Al_2O_3 (r-cut) samples, the corresponding Bragg reflections were observed at nearly the same angular positions. These measurements yield the orientation of epitaxial VO_2 films on the r-cut Al_2O_3 as: $\mathbf{b}_{M1}||[100]_{Al2O3}$, $\mathbf{c}_{M1}||[02\bar{1}]_{Al2O3}$, where $\mathbf{b}_{M1}$ and $\mathbf{c}_{M1}$ are crystallographic axes of VO_2 in the monoclinic M1 phase [2-4]. (100)VO_2 plane in M1 phase is parallel to the substrate plane (012)Al_2O_3 (r-cut). Along with their well-defined orientation, the epitaxial VO_2 films show no detectable twinning [3]. Large lattice mismatch between VO_2 and r-cut sapphire results in misfit strains of 4.0% and -5.1% along $\mathbf{b}_{M1}$ and $\mathbf{c}_{M1}$ axes of VO_2, respectively [4].

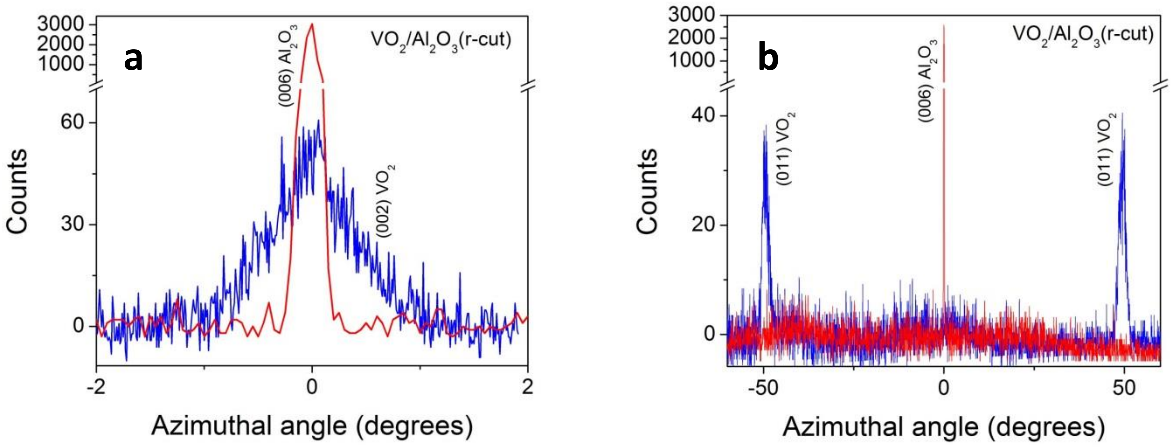

Supplementary Figure 2. Azimuthal scans of X-ray diffraction signal for 35 nm VO_2 film on Al_2O_3 (r-cut) substrate. **a** XRD from (002)VO_2 and (006)Al_2O_3 planes; **b** XRD from (011)VO_2 and (006)Al_2O_3 planes.

Upon the phase transition, the unit cell parameters of VO_2 change from a_{M1}=5.7517 Å, b_{M1}=4.5378 Å, c_{M1}=5.3825 Å in the monoclinic phase (monoclinic angle β=122.646°) [5] to a_r=4.53 Å, b_r=4.53 Å, c_r=2.869 Å in the rutile phase [6]. For the epitaxial film on r-cut sapphire, the orientation of the VO_2 lattice changes upon the phase transition from [200] in the monoclinic phase to [011] in the rutile phase [7].

In order to measure the thicknesses of the VO_2 films we used a Horiba Scientific Uvisel 2 ellipsometer and the Delta Psi software. To account for the films' roughness in the model, a 50% VO_2/air layer was included on top of the continuous VO_2 layer on top of the Al_2O_3 substrate. The obtained thicknesses of the VO_2 layers, including half the roughness, are 100±6 nm and 36±4 nm. Thus, in the following we use values of 100 and 35 nm as thicknesses of the two films.

Supplementary Note 2. Temperature of the phase transition in the VO_2 films

In order to determine the temperature at which the studied VO_2 films demonstrate the phase transition, their reflectivity as a function of temperature was measured at the laser wavelength λ=1028 nm (Supplementary Figure 3). The transition temperatures for 100 nm and 35 nm films are found to be 323 K and 315 K, respectively, which are lower than the bulk transition temperature 340 K [8]. This is a known effect of a built-in strain. In VO_2 films on r-cut Al_2O_3 built-in strain relaxes rapidly as the film thickness exceeds ~80 nm [9]. This accounts for the smaller discrepancy between the actual and the bulk phase transition temperatures found in the 100 nm VO_2 film.

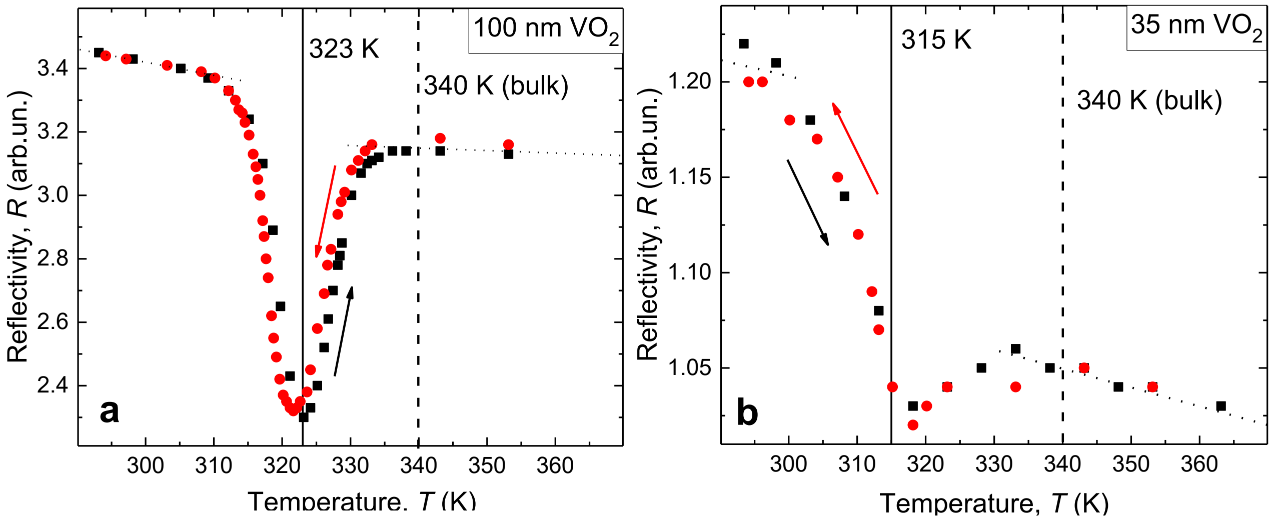

Supplementary Figure 3. Temperature dependence of the reflectivity R at λ=1028 nm of the (**a**) 100 nm and (**b**) 35 nm VO_2 films. Black squares and red circles show reflectivity changes upon heating and cooling cycles, respectively. Vertical solid lines mark the transition temperatures T_c. The value for T_c=340 K for the bulk VO_2 is also marked with the vertical dashed line.

Supplementary Note 3. Threshold and saturation optical fluences of PIPT in VO_2 films

The photo-induced counterpart of insulator-to-metal and structural phase transition in VO_2 is characterized by a threshold W_T and saturation W_S laser pulse fluences. W_T is a minimal fluence sufficient to induce PIPT in any part of the film, while W_S marks the fluence capable of transforming all of the VO_2 volume within the laser spot into its metallic phase.

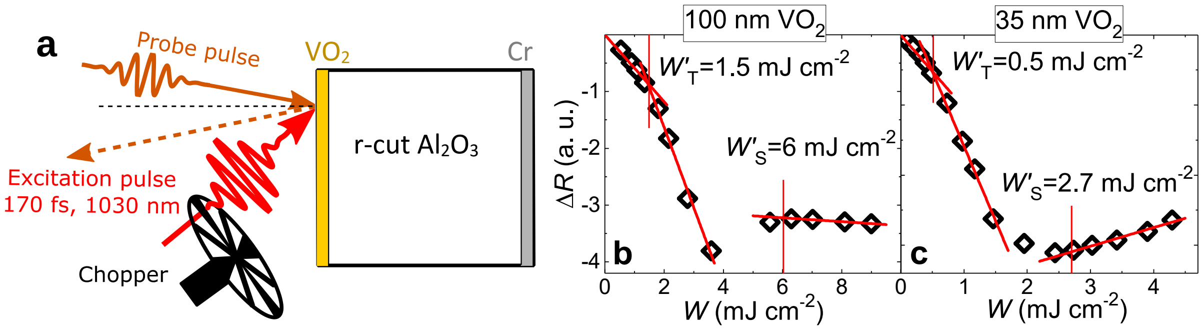

Supplementary Figure 4: **a** Optical pump-probe setup used to obtain W_T and W_S. **b**,**c** Fluence dependence of the reflectivity change ΔR as obtained at 1 ps time delay (**b**) for the 100 nm VO_2 film, and (**c**) for the 35 nm VO_2 film. Threshold W'_T and saturation W'_S fluences and the way to obtain them are shown.

W_T and W_S values were measured using a conventional optical pump-probe setup (Supplementary Figure 4(a)) with excitation and probe laser pulses incident on VO_2 directly. The

same laser source as in the main experiments was used with wavelength of 1028 nm, repetition rate of 5 kHz, and pulse duration of 170 fs. We measured the change of the probe intensity reflected from VO_2 as a function of the time delay between the pump and probe pulses at different excitation pulse fluences W. These changes are directly proportional to the changes of the VO_2 reflectivity ΔR as a result of laser excitation. The dependence $\Delta R(W)$ obtained at time delay 1 ps is shown in Supplementary Figure 4(b,c) for the two studied films. This particular time delay was also used by other researchers to evaluate the threshold and saturation values of the PIPT in VO_2 in order to derive parameters of the non-thermal stage of the PIPT [8].

The threshold fluence W'_T is the lowest fluence found at which the abrupt change of slope of the dependence $\Delta R(W)$ occurs, and saturation fluence W'_S is determined as W above which the slope does not change. We obtained the values W'_T=1.5 mJ cm^{-2}, W'_S=6 mJ cm^{-2} for the 100 nm VO_2 film, and W'_T=0.5 mJ cm^{-2}, W'_S=2.7 mJ cm^{-2} for the 35 nm film (Supplementary Figure 4(b,c)).

In order to apply the determined values of W'_T and W'_S to the main experiments with optical pulses exciting strain in VO_2 films (Fig. 2 of the main text), we have to take into account the different angles of incidence used in the optical pump probe experiment (60°) and in the main experiments (0°). Because of p-polarization of pump, higher fraction of the optical pulse energy density is deposited on the VO_2 film when the angle of incidence is 60°. Therefore, the threshold and saturation fluences are higher in the main experiments, where the pump beam is incident normally on the VO_2 layer. Recalculated values shown on Fig. 2 of the main text are W_T=2 mJ cm^{-2}, W_S=8 mJ cm^{-2} for the 100 nm VO_2 film, and W_T=0.7 mJ cm^{-2}, W_S=3.6 mJ cm^{-2} for the 35 nm film.

Supplementary Note 4. Pump volume energy density absorbed in VO_2 films

To determine the pump volume energy density J absorbed in the VO_2 layer at each incident fluence W, we used the expression for the normalized absorption $A/P=(P-R-T)/P$, where P, R, and T are the incident, reflected, and transmitted average pump power, respectively. The results for both samples and at temperatures both below and above the transition temperature are shown in Supplementary Figure 5.

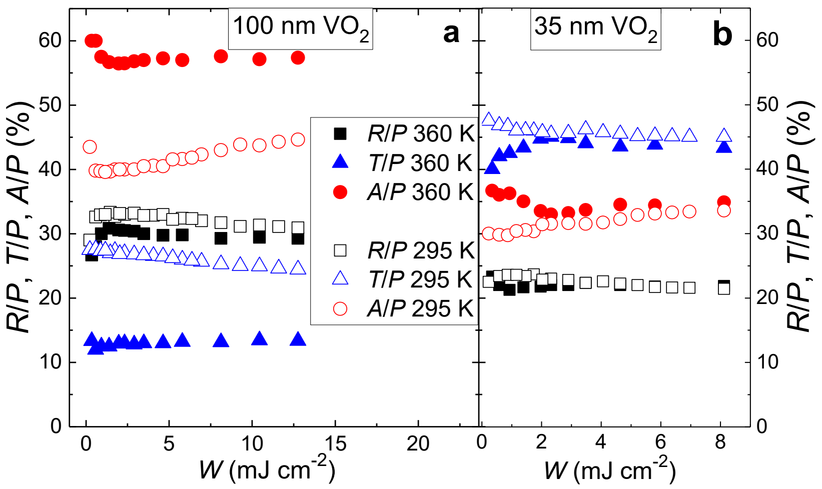

Supplementary Figure 5. Normalized reflectivity R/P, transmission T/P, and absorption, A/P as a function of the pump fluence W measured at λ=1028 nm for (**a**) 100 nm VO_2 and (**b**) 35 nm VO_2. Open and closed symbols are the data obtained at room temperature and at T=355 K, respectively.

Supplementary Note 5. Individual contributions to strain from insulating and metallic phases upon laser-induced PIPT

We consider three intervals of the optical excitation density J:

(i) $J<J_T$. No PIPT occurs and the whole excited material gives rise to the lattice heating governed by the parameters of the insulating VO_2. Taking into account possible distribution of J_T with a dispersion σ_T, the weight of this "fully insulating" contributions to the thermoelastic and deformation potential mechanisms are described by $0.5 \cdot \left[1 - \mathrm{erf}\frac{J-J_T}{\sqrt{2}\sigma_T}\right]$.

(ii) $J_T<J<J_S$. As J exceeds J_T, the fraction of the material in the insulating phase decreases with excitation energy and reaches zero at $J=J_S$. We calculate the weight of the contributions from the insulating phase as $0.5 \cdot \left[1 - \left(\mathrm{erf}\frac{J-J_T}{\sqrt{2}\sigma_T}\right)\left(\mathrm{erf}\frac{J-J_0}{\sqrt{2}\sigma_0}\right)\right]$. Alternatively, the fraction of metallic phase grows with the increase of J with the weight $0.5 \cdot \left[1 - \left(\mathrm{erf}\frac{J-J_S}{\sqrt{2}\sigma_S}\right)\left(\mathrm{erf}\frac{J-J_0}{\sqrt{2}\sigma_0}\right)\right]$.

(iii) $J \geq J_S$. The whole excited material gives rise to the lattice heating governed by the parameters of the metallic phase emerged due to PIPT, and the weight of this "fully metallic" contribution to the lattice heating is described by $0.5 \cdot \left[1 + \mathrm{erf}\frac{J-J_S}{\sqrt{2}\sigma_S}\right]$.

We note that in calculating the thermoelastic contribution we use thermodynamic and elastic parameters for the phase (insulating or metallic) established at a time before the structural PIPT takes place [8]. This means that the whole energy absorbed by VO_2, apart from the energy spent on structural PIPT, is included in the calculation of temperature rise and generated strain. The values for these energies are equal to: J at $J<J_T$; J_T at $J_T<J<J_S$; and $J-\Delta J$ at $J \geq J_S$.

Supplementary Figure 6(a) shows the lattice temperature due to the laser-induced heating which originates from the fractions of material remaining in the insulating phase (blue dotted line), and the material which underwent PIPT (red dashed line). The resulting temperature is shown by the black solid line. The initial temperature of the sample is 295 K. In the calculations we used the values $J_T=0.75\cdot10^8$ J m^{-3}, $J_S=3.16\cdot10^8$ J m^{-3}, $\sigma_0=(J_S-J_T)/4$, $\sigma_T=\sigma_S=(J_S-J_T)/40$. ΔJ~$2.2\cdot10^8$ J m^{-3} is chosen to ensure the total heating to the transition temperature 323 K achieved at $J=J_S$. Supplementary Figure 6(b) shows the excitation energy dependence of the strain generated via the thermoelastic effect by insulating and metallic fraction accordingly to the calculated temperature increase.

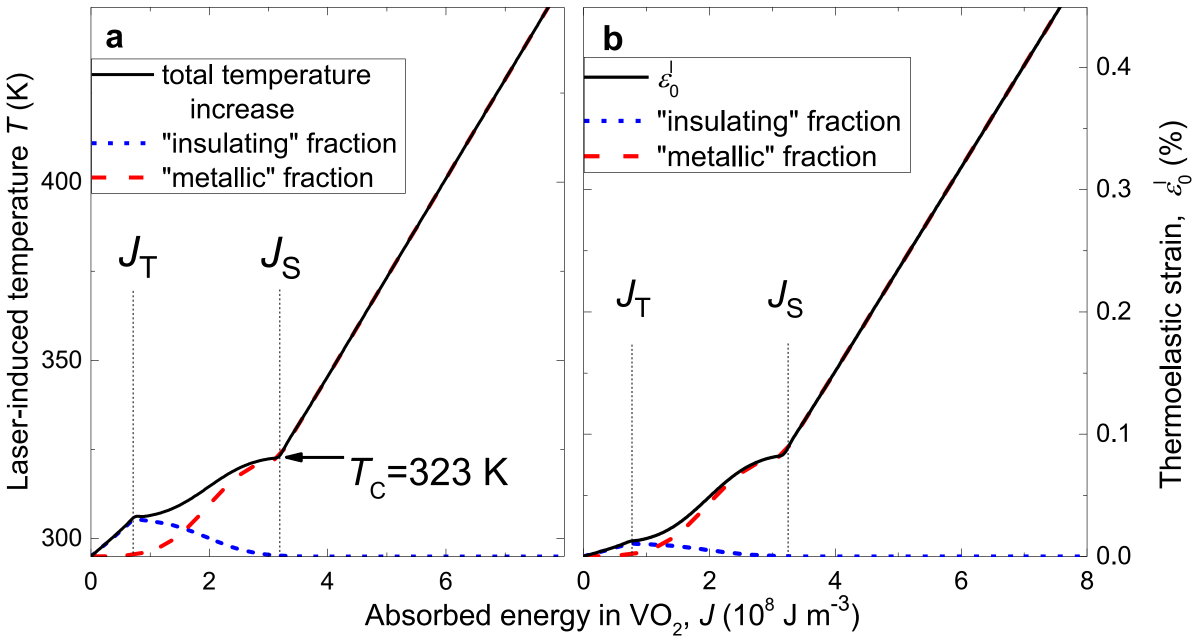

Supplementary Figure 6. Calculated absorbed energy dependence of (**a**) the lattice temperature $T(J)$ and (**b**) the related thermoelastic contribution to the laser-generated strain $\varepsilon_0^l(J)$ resulting from the laser excitation of VO_2 in the insulating phase at 295 K. The blue dotted line shows the contribution of the material remaining in the insulating phase after the excitation, red dashed line - from the fraction of the material which underwent PIPT. The black solid line is the total temperature increase and the thermoelastic contribution to the strain.

Supplementary Note 6. Error analysis of the simulation of strain pulse propagation and detection

In the model of strain pulse generation used to build the calibration curve $\tau \rightarrow \varepsilon_0$ the main uncertainty arises from elastic parameters of the VO_2 film. The parameters of the r-cut sapphire responsible for the strain pulse evolution upon propagation are known [10] with exception of viscosity. The latter is assumed to be isotropic and was taken equal to the value for c-cut Al_2O_3 [11]. In order to simulate the strain pulse injected from the VO_2 film into sapphire one needs to know mass density, light penetration depth, longitudinal sound velocity, and thickness of VO_2. Mass density and sound velocity s_{VO2} determine reflections of the strain pulse at the interface with the sapphire substrate, while the light penetration depth and sound velocity determine initial strain pulse duration which has a pronounced impact on simulation results.

Penetration depth was calculated with optical parameters of bulk VO_2 [12] which for the studied VO_2 films yielded the same absorption as measured experimentally (Supplementary Figure 5). The sound velocity was calculated using bulk VO_2 elastic constants obtained theoretically in [13]. For the error estimation we considered a possible uncertainty in the latter value.

The lower limit for the sound velocity was determined by the shape of the photoelastic response (see Figs. 2a,b of the main text), namely the relative amplitude of sharp negative peaks.

The decrease in the VO_2 sound velocity results in one of the peaks being smaller than the other. Taking into account experimental uncertainty and noise, we were able to estimate thus the lower limit for the VO_2 sound velocity. The higher limit for s_{VO2} was taken to be 11500 m s^{-1} [14] as it is the highest value ever reported in literature. Though it was reported for the metallic phase of VO_2 only, we took the same value for the dielectric phase of VO_2 as a reasonable estimation. We note here, that the higher the sound velocity, the shorter the initial strain pulse corresponding to a particular signal duration τ, which leads to even higher estimate for the strain generated in VO_2 film for a particular absorbed energy.

This led us to the following range of sound velocities in VO_2 film on r-cut sapphire:
for insulating phase: 9300 m s^{-1} – 11500 m s^{-1},
for metallic phase: 7500 m s^{-1} – 11500 m s^{-1}.
Using these values, we calculated the uncertainty range for the $\tau \rightarrow \varepsilon_0$ calibration shown in Fig. 1e of the main text as shaded areas. This uncertainty range led to the margins of the strain value derived from the experimental data using this calibration curve (Fig. 3a,b).

The uncertainty in the calibration curve yields the margins for the $\varepsilon_0(J)$ (Figs. 3a,b of the main text), i.e. this curve acquires different slopes while keeping its shape. The latter would yield different value of deformation potential Ξ_i=-4±0.7 eV. The conclusion about the presence and a character of the non-thermal contribution to the strain generation would remain the same, with the uncertainty of its absolute value being $\varepsilon_0^{\mathrm{pt}} = +0.45^{+0.19}_{-0.05}\%$.

Supplementary References

[1] S. Lysenko, F. Fernández, A. Rúa, and H. Liu, Ultrafast light scattering imaging of multi-scale transition dynamics in vanadium dioxide, *J. Appl. Phys.*, **114**, 153514 (2013).

[2] M. Borek, F. Qian, V. Nagabushnam, and R. K. Singh, Pulsed laser deposition of oriented VO2 thin films on R-cut sapphire substrates, *Appl. Phys. Lett.*, **63**, 3288 (1993).

[3] S. Lysenko, V. Vikhnin, F. Fernandez, A. Rua, and H. Liu, Photoinduced insulator-to-metal phase transition in VO2 crystalline films and model of dielectric susceptibility, *Phys. Rev. B*, **75**, 075109 (2007).

[4] T.-H. Yang, R. Aggarwal, A. Gupta, H. Zhou, R. J. Narayan, and J. Narayan, Semiconductor-metal transition characteristics of VO_2 thin films grown on c- and r-sapphire substrates, *J. Appl. Phys.*, **107**, 053514 (2010).

[5] J. M. Longo and P. Kierkegaard, A Refinement of the Structure of VO_2, *Acta Chem. Scand.*, **24**, 420 (1970).

[6] S. Westman, Note on a Phase Transition in VO2, *Acta Chem. Scand.*, **15**, 217 (1961).

[7] Y. Zhao, J. H. Lee, Y. Zhu, M. Nazari, C. Chen, H. Wang, A. Bernussi, M. Holtz, and Z. Fan, Structural, electrical, and terahertz transmission properties of VO_2 thin films grown on c-, r-, and m-plane sapphire substrates, *J. Appl. Phys.*, **111**, 053533 (2012).

[8] D. Wegkamp and J. Stähler, Ultrafast dynamics during the photoinduced phase transition in VO2, *Progr. Surf. Sci.*, **90,** 464–502 (2015).

[9] S. Lysenko, F. Fernández, A. Rúa, J. Aparicio, N. Sepúlveda, J. Figueroa, K. Vargas, and J. Cordero, Light scattering by epitaxial VO_2 films near the metal-insulator transition point, *J. Appl. Phys.*, **117**, 184304 (2015).

[10] J. M. Winey, Y. M. Gupta, D. E. Hare, R-axis sound speed and elastic properties of sapphire single crystals, *J. Appl. Phys.*, **90**, 3109 (2001).

[11] P. J. S. van Capel and J. I. Dijkhuis, Time-resolved interferometric detection of ultrashort strain solitons in sapphire, *Phys. Rev. B,* **81**, 144106 (2010).
[12] H. W. Verleur, A. S. Barker, Jr., C. N. Berglund, Optical Properties of VO_2 between 0.25 and 5 eV, *Phys. Rev.*, **172**, 788 (1968).
[13] H. Dong, H. Liu, Elastic properties of VO_2 from first-principles calculation, *Solid State Commun.*, **167**, 1 (2013).
[14] E. Abreu, S. N. G. Corder, S. J. Yun, S. Wang, J. G. Ramírez, K. West, J. Zhang, S. Kittiwatanakul, I. K. Schuller, J. Lu, S. A. Wolf, H.-T. Kim, M. Liu, and R. D. Averitt, Ultrafast electron-lattice coupling dynamics in VO_2 and V_2O_3 thin films, *Phys. Rev. B,* **96**, 094309 (2017).
